# Piezo-Type Mechanosensitive Ion Channel Component 1 (PIEZO1) as a Potential Prognostic Marker in Renal Clear Cell Carcinoma

**DOI:** 10.3390/ijms26146598

**Published:** 2025-07-09

**Authors:** Paulina Antosik, Martyna Szachniewicz, Michał Baran, Klaudia Bonowicz, Dominika Jerka, Ewelina Motylewska, Maciej Kwiatkowski, Maciej Gagat, Dariusz Grzanka

**Affiliations:** 1 Department of Clinical Pathomorphology, Faculty of Medicine, Collegium Medicum in Bydgoszcz, Nicolaus Copernicus University in Torun, 85-094 Bydgoszcz, Poland; martynaszachniewicz@wp.pl (M.S.); 1michalbaran@gmail.com (M.B.); d_grzanka@cm.umk.pl (D.G.); 2 Department of Histology and Embryology, Collegium Medicum in Bydgoszcz, Nicolaus Copernicus University in Torun, 85-092 Bydgoszcz, Poland; klaudia.bonowicz@cm.umk.pl (K.B.); dominika.jerka@cm.umk.pl (D.J.); mgagat@cm.umk.pl (M.G.); 3 Department of Morphological and Physiological Sciences, Faculty of Medicine, Collegium Medicum, Mazovian Academy in Płock, 09-402 Płock, Poland; 4 Department of Immunoendocrinology, Chair of Endocrinology, Medical University of Lodz, 92-213 Lodz, Poland; ewelina.motylewska@umed.lodz.pl; 5 Department of Urology and Urological Oncology, Multidisciplinary Hospital of Ludwik Blażek, 88-100 Inowrocław, Poland; maciej_kwiatkowski@yahoo.com

**Keywords:** PIEZO1, renal clear cell carcinoma, prognostic factor

## Abstract

Clear cell renal cell carcinoma (ccRCC) is the most common histological subtype of kidney cancer and is often diagnosed at advanced stages. PIEZO1, a mechanosensitive ion channel, has been implicated in cancer progression, but its prognostic relevance in ccRCC remains unclear. This study aimed to evaluate the expression pattern of PIEZO1 in ccRCC and its association with clinicopathological characteristics and patient survival. Immunohistochemical analysis was performed on formalin-fixed, paraffin-embedded tumor tissues from 111 patients with ccRCC, along with 23 matched peritumoral non-cancerous tissues. Protein expression was quantified using the H-score system. Associations with tumor grade, staging, and overall survival (OS) were analyzed. mRNA expression data were retrieved from The Cancer Genome Atlas (TCGA) to validate the protein-level findings. Functional enrichment and pathway analyses were conducted to explore the biological context of PIEZO1-related gene expression. PIEZO1 showed predominantly cytoplasmic localization, with significantly lower expression in tumor tissues compared to adjacent non-malignant tissue (*p* < 0.0001). High PIEZO1 expression was correlated with higher tumor grade (*p* = 0.0147) and shorter OS (*p* = 0.0047). These findings were confirmed at the mRNA level in the TCGA cohort. Multivariate Cox regression analysis identified PIEZO1 as an independent prognostic factor for OS. In conclusion, PIEZO1 may serve as a clinically relevant biomarker in ccRCC. Its overexpression is associated with more aggressive tumor characteristics and poor prognosis, underscoring the need for further investigation into its functional role and potential as a therapeutic target.

## 1. Introduction

Renal cell carcinoma (RCC) is the 14th most common cancer worldwide, with over 430,000 new diagnoses and over 179,000 RCC deaths in the year 2020 [1]. It occurs more often in men and in the past 2 decades there has been a 2% yearly increase in RCC incidence worldwide [2]. It is also commonly found at a late stage, with up to 17% of patients having distant metastasis at diagnosis [3,4]. Clear cell renal cell carcinoma (ccRCC) is the most common subtype (70–80%) and accounts for the majority of RCC-related deaths, making it the most aggressive form [2,5,6]. About 30% of ccRCC patients develop distant metastasis, including those after nephrectomy [5]. The identification of novel diagnostic markers, prognostic indicators, and therapeutic targets remains crucial for improving survival outcomes in patients with ccRCC.

PIEZO1 is recognized as a mechanosensitive ion channel that responds to mechanical stimuli such as stretching, compression, and shear stress [7]. It causes an influx of calcium ions, which influence numerous physiological processes in the cell [8]. This protein is encoded by the *PIEZO1* gene, located in the locus 16q24.3 [9]. PIEZO1 is a trimeric transmembrane protein, with three identical subunits, forming an ion channel in the center and three blades curved outwards. These blades likely respond to membrane distortion by pushing open the central pore [10]. PIEZO1 is commonly expressed in the skin, bladder, lungs, digestive tract, and blood vessel lining, and it is less expressed in the skeletal muscles and cerebellum [8,10]. Growing evidence indicates that PIEZO1 functions as a key regulator of several physiological processes, including bone remodeling, erythrocyte volume regulation, iron metabolism, neurogenesis, cell migration, and embryonic development [11,12,13,14,15,16,17]. Overexpression of PIEZO1 has been reported in multiple human cancers and is commonly associated with unfavorable prognosis [18]. Such overexpression has been observed in breast, colorectal, prostate, gastric, oral squamous cell carcinoma, synovial sarcoma, glioma, and osteosarcoma cancers [18,19,20,21,22,23,24,25,26]. PIEZO1 has been implicated in the regulation of tumor cell proliferation through activation of the PI3K/AKT/mTOR signaling pathway, promotion of angiogenesis via HIF-1α upregulation, and facilitation of processes such as migration, invasion, metastasis, and epithelial–mesenchymal transition (EMT), while also reducing apoptosis [27,28]. Notably, in lung cancer, PIEZO1 overexpression has been associated with improved overall survival, suggesting a divergent, potentially tumor-suppressive role in this context [29].

In this study, we aimed to investigate the expression pattern and potential clinical significance of PIEZO1 in ccRCC. The initial phase involved the immunohistochemical analysis of PIEZO1 expression in ccRCC tissues. Protein expression levels were evaluated based on our own immunohistochemical data, while mRNA expression was assessed using transcriptomic datasets obtained from The Cancer Genome Atlas (TCGA). Both types of data were analyzed in relation to clinicopathological parameters and overall survival (OS) of patients. Our analyses aimed to characterize the transcriptional landscape of PIEZO1 and explore its potential regulatory networks in tumor biology. Functional enrichment and pathway analyses were conducted to identify biological processes, molecular functions, and cellular components associated with PIEZO1 expression. By examining gene expression patterns and co-expression networks, we sought to uncover potential oncogenic interactions that may contribute to tumor progression and impact patient prognosis. By integrating experimental and bioinformatic approaches, this study aims to define the prognostic value of PIEZO1 expression in ccRCC and to provide novel insights into its role in renal tumorigenesis.

## 2. Results

### 2.1. PIEZO1 Expression Patterns in ccRCC and Adjacent Non-Tumor Tissues

**Figure 1:**
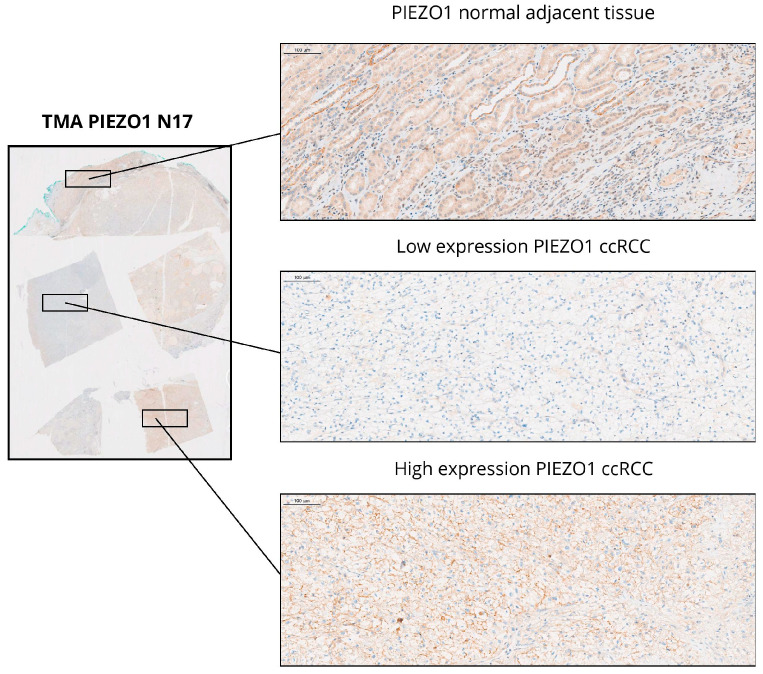
PIEZO1 expression in ccRCC tissues in our cohort. The TMA slide N17 (×4) contained 5 tumor and normal adjacent tissue fragments that were immunostained with anti-PIEZO1 antibody. Representative magnification (×10) of PIEZO1 immunostaining in three fragments shows the cytoplasmic localization of this protein in normal adjacent tissue, and low- and high-expression PIEZO1 in ccRCC.

**Figure 2:**
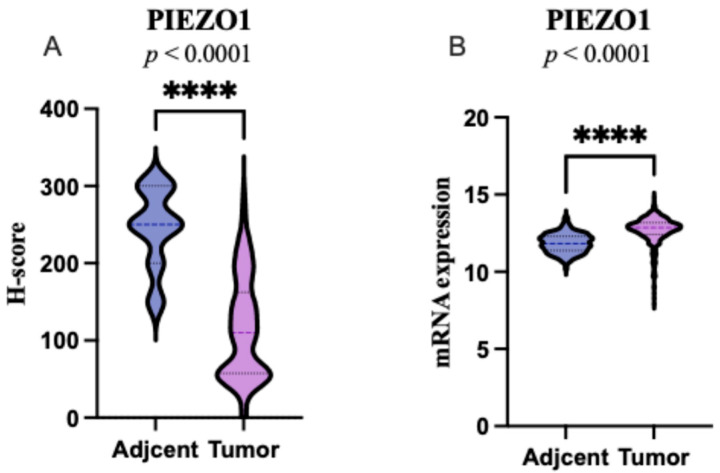
Expression of PIEZO1 in clear cell renal cell carcinoma compared to adjacent tissue. Boxplot graphs of (PIEZO1 expression in tumor tissue *(n* = 111) and histological normal tissue adjacent to the tumor (*n* = 23) in our cohort (**A**); *PIEZO1* expression levels in tumor tissue (*n* = 504) and adjacent normal tissue (*n* = 129) based on RNA-Seq data from the TCGA (**B**). Protein levels were assessed using the H-score method (range: 0–300), which reflects both staining intensity and the proportion of positively stained cells. The width of the violin at each expression level shows the data density, the horizontal line inside marks the median, and each dot represents an individual sample.

Immunohistochemical analysis revealed cytoplasmic localization of PIEZO1 in both ccRCC tissues and adjacent non-tumor kidney samples. Strong cytoplasmic staining of PIEZO1 was observed in the epithelial cells of distal renal tubules within non-tumorous peritumoral tissue. In tumor samples, more intense PIEZO1 expression was frequently noted in the peripheral part of the tumor. Representative immunohistochemical images are shown in Figure 1. Among the 111 tumor samples analyzed, low PIEZO1 expression was identified in 59 cases (53.15%), while high expression was observed in 52 cases (46.85%). Overall, PIEZO1 expression was significantly reduced in ccRCC tissues compared to adjacent non-tumor kidney tissues (*p* < 0.0001; Figure 2A). The RNA-Seq data from the TCGA showed that the PIEZO1 expression was significantly increased in ccRCC tumor tissues compared to the matched adjacent normal tissue (*p* < 0.0001; Figure 2B).

### 2.2. Association with the Clinicopathological Characteristics

PIEZO1 expression status was significantly associated with clinicopathological characteristics, including tumor grade (*p* = 0.0147; Table 1). ccRCCs with increasing tumor grade more frequently had PIEZO1 high- than PIEZO1 low-expression. No other associations were found between PIEZO1 and investigated clinicopathological parameters. In the TCGA cohort (Table 1) no significant associations were found between PIEZO1 expression and clinicopathological features, including age, sex, grade, pT status, pN, and stage.

### 2.3. Association with the Clinical Outcome

**Figure 3:**
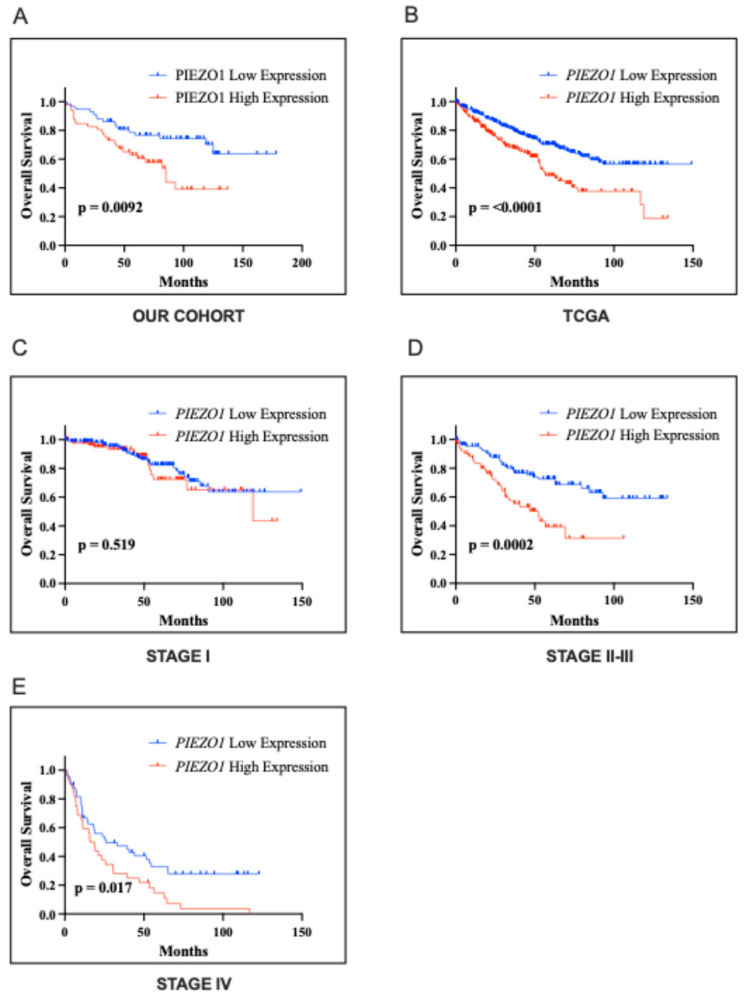
Effect of PIEZO1 and mRNA PIEZO1 expression on survival in our cohort and The Cancer Genome Atlas (TCGA) data portal cohort. Cases were divided into low- and high-expression groups according to the optimal cut-off point (our cohort Cp = 110, TCGA cohort Cp = 13.01) determined by the Evaluate Cutpoints software (R version 3.4.1). Kaplan–Meier curves comparing low and high PIEZO1 expression in our cohort (*n* = 111) (**A**) and low and high expression of PIEZO1 in the TCGA cohort (*n* = 504) (**B**). Kaplan–Meier curves comparing low and high expression of PIEZO1 in stage I subgroup (**C**), stage II–III (**D**), and stage IV (**E**). *p*-values were calculated by the log-rank test.

In Kaplan–Meier survival analysis of our cohort, PIEZO1 high-expression was significantly associated with shortened median OS (85 months) compared with PIEZO1-low (undefined median; *p*  =  0.0092; Figure 3A). As presented in Table 2, statistically significant HRs were found for PIEZO1 (HR 2.13, 95% CI 1.14–3.98), sex (HR 2.66, 95% CI 1.31–5.43), grade (HR 2.88, 95% CI 1.44–5.75), pT status (HR 3.59, 95% CI 1.84–7.00), pN status (HR 17.17, 95% CI 3.72–79.14), and lymphovascular invasion (LVI) (HR 6.35, 95% CI 2.23–18.08). In a subsequent multivariable analysis, all of these remained independent prognostic factors for OS (Table 2; all *p*  <  0.05).

In Kaplan–Meier survival analysis of the TCGA cohort, *PIEZO1* high-expression was significantly associated with shortened median OS (56 months) compared with PIEZO1 low expression (undefined median; *p* < 0.0001; Figure 3B), as well as across stage I (119 months vs. undefined median; *p* = 0.519; Figure 3C), stage II-III (52 months vs. undefined median; *p* = 0.0002; Figure 3D), and stage IV (17 months vs. 26 months median; *p* = 0.017; Figure 3E).

### 2.4. In Silico Analysis of Functional Pathways Linked to PIEZO1-Correlated Genes

The top genes positively and negatively correlated with *PIEZO1* were identified using Spearman’s correlation. The genes *NLRPI1* and *TECPR1* showed the highest positive correlation with *PIEZO1*, with Spearman’s correlation coefficients of 0.59 and 0.582, respectively. The genes *TSG101* and *SDHD* showed the highest negative correlation with *PIEZO1*, with Spearman’s correlation coefficients of −0.55 and −0.54, respectively. These findings indicate a strong relationship between *PIEZO1* and these genes, suggesting potential shared regulatory mechanisms or functional roles in the studied context (Table 3 and Table 4).

**Figure 4:**
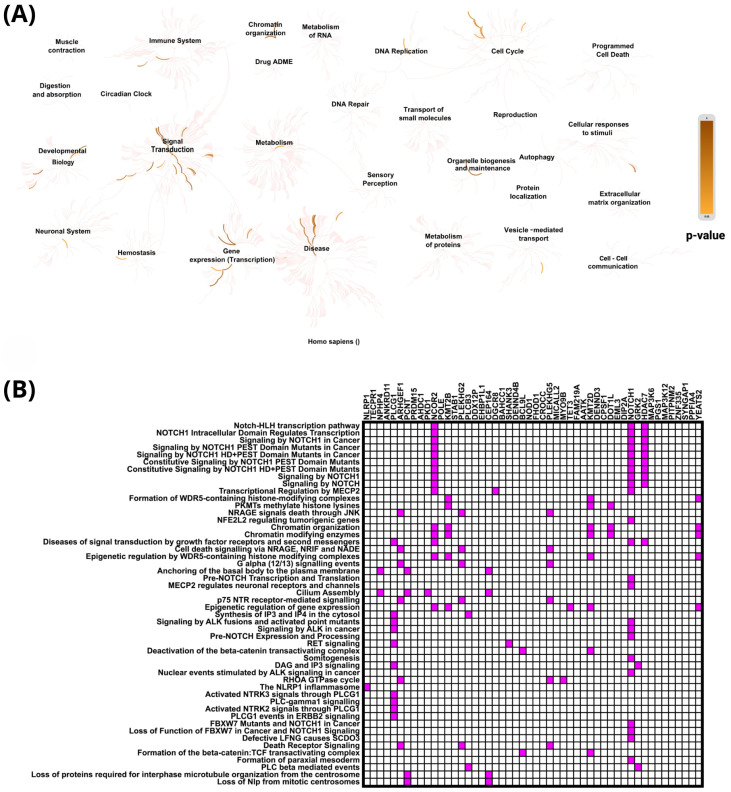
Functional enrichment analysis based on the TCGA dataset. The top 50 genes and reactome pathways positively correlated with *PIEZO1* expression (**A**,**B**).

Reactome pathway analysis revealed that genes exhibiting positive correlation with PIEZO1 in ccRCC were significantly enriched in key biological processes such as signal transduction, cell cycle regulation, and gene expression (transcription) (Figure 4A). Among the most significantly associated pathways, the top three were the Notch-HLH transcription pathway (*p* = 3.11 × 10^−6^), NOTCH1 Intracellular Domain Regulates Transcription (*p* = 5.00 × 10^−5^), and Signaling by NOTCH1 in Cancer (*p* = 9.76 × 10^−5^). Importantly, the analysis demonstrated that the first nine most enriched pathways were all closely linked to Notch signaling (Figure 4B). Notably, NOTCH1 appeared most frequently among the top enriched pathways, further underscoring its central role in the regulatory mechanisms associated with PIEZO1 expression.

**Figure 5:**
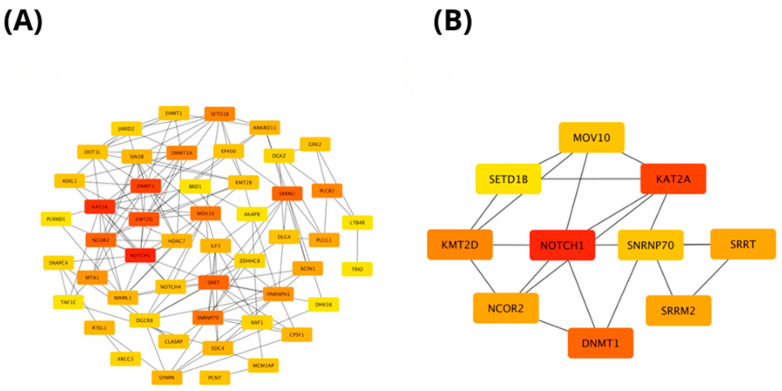
PPI network of genes positively correlated with *PIEZO1*. The interaction network includes the top 50 genes positively associated with *PIEZO1*, where each node represents a gene, and the color intensity corresponds to its connectivity within the network (**A**). The top 10 hub genes within the *PIEZO1*-related network were identified using the CytoHubba plugin in Cytoscape and are shown with a red-to-yellow gradient, where deeper red denotes higher connectivity and stronger functional relevance (**B**).

The protein–protein interaction (PPI) networks of genes positively correlated with *PIEZO1* in ccRCC were constructed using STRING (https://string-db.org; accessed on 2 March 2025) and visualized in Cytoscape software (version 3.10.3, Cytoscape Consortium, San Diego, CA, USA). The resulting network comprised 50 nodes, demonstrating significant interaction enrichment (PPI enrichment *p*-value < 1.0 × 10^−16^). As shown in Figure 5A, several genes exhibited strong functional associations with *PIEZO1*, forming a densely interconnected network. To identify key regulatory elements within this network, hub gene analysis was conducted using the cytoHubba plugin in Cytoscape software (version 3.10.3, Cytoscape Consortium, San Diego, CA, USA), ranking genes according to their degree of connectivity. The top-ranked hub genes, indicated in red, reflect those with the highest interaction degrees and potential functional relevance. Figure 5B presents a focused view of these hub genes, among which *NOTCH1*, *KAT2A*, *DNMT1*, and *KMT2D* showed the highest scores. These genes may play crucial roles in *PIEZO1*-related signaling pathways and could contribute to the molecular mechanisms underlying ccRCC progression, warranting further functional characterization.

**Figure 6:**
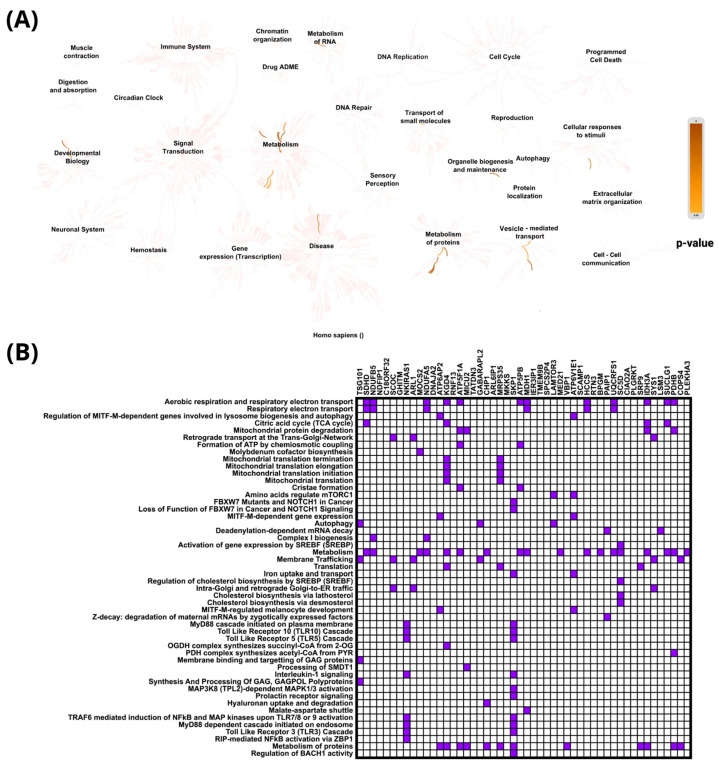
PPI network of genes negatively correlated with *PIEZO1*. The network includes the top 50 negatively associated genes, with node color indicating connectivity (**A**). The 10 most connected hub genes, identified using CytoHubba, are shown with a red-to-yellow gradient, where deeper red reflects stronger interaction relevance (**B**).

Reactome pathway analysis of genes negatively correlated with *PIEZO1* in clear cell ccRCC indicated significant enrichment in metabolic processes, particularly those related to general metabolism, protein metabolism, and RNA metabolism (Figure 6A). The most significantly overrepresented pathways included aerobic respiration and respiratory electron transport (*p* = 4.36 × 10^−9^), respiratory electron transport (*p* = 7.09 × 10^−5^), and regulation of MITF-M-dependent genes involved in lysosome biogenesis and autophagy (*p* = 1.43 × 10^−4^). Additionally, notable enrichment was observed in pathways such as the citric acid cycle (TCA cycle) and mitochondrial protein degradation, further highlighting the involvement of mitochondrial and metabolic functions (Figure 6B). These findings suggest that reduced *PIEZO1* expression may be linked to compensatory metabolic reprogramming in ccRCC tumors. This shift in cellular metabolism likely reflects altered bioenergetic demands and disrupted proteostasis within the tumor microenvironment, potentially facilitating cancer progression. Among the negatively correlated genes, *SKP1* emerged as a representative example, being associated with several of the enriched metabolic pathways.

**Figure 7:**
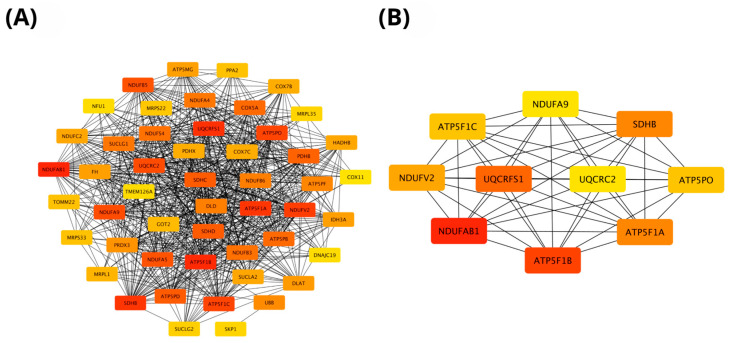
PPI network of genes negatively correlated with *PIEZO1*, including the top 50 negatively correlated genes (**A**) and the top 10 hub genes identified in the network (**B**).

The PPI network of genes negatively correlated with *PIEZO1* in ccRCC also revealed 50 nodes with strong interaction enrichment (*p* < 1.0 × 10^−16^), similarly to the network of positively correlated genes. As shown in Figure 7A, genes involved in mitochondrial function and metabolism formed a highly interconnected network. Hub gene analysis using cytoHubba identified *NDUFAB1*, *ATP5F1B*, *UQCRFS1*, and *SDHB* as top-ranked nodes (Figure 7B), suggesting their central role in *PIEZO1*-associated metabolic regulation. These findings highlight a potential link between low *PIEZO1* expression and mitochondrial activity in ccRCC.

**Figure 8:**
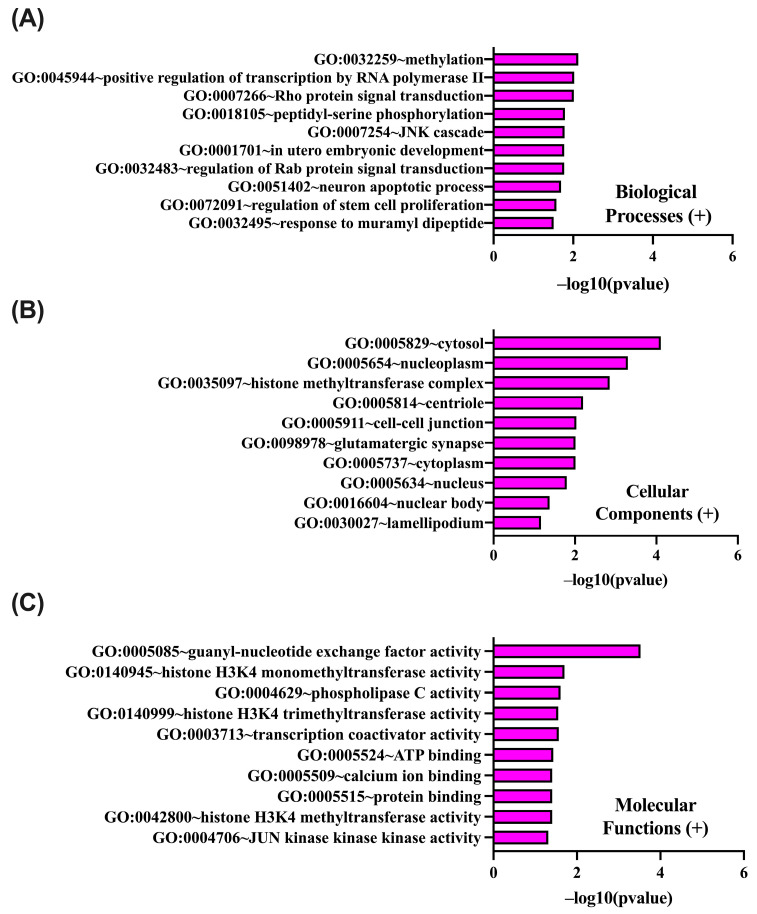
DAVID GO analysis of genes positively correlated with PIEZO1, categorized into biological process (BP) (**A**), cellular component (CC) (**B**), and molecular function (MF) (**C**). The top 10 GO terms are shown for each category, with *p*-values calculated and ranked based on −log10 (*p*-value).

Gene Ontology (GO) enrichment analysis was performed for genes positively correlated with PIEZO1 in ccRCC using the DAVID platform (https://david.ncifcrf.gov; accessed on 5 March 2025). The results were categorized into biological processes (BP), cellular components (CC), and molecular functions (MF) (Figure 8A–C). Among BP terms, the most significantly enriched were methylation, positive regulation of transcription by RNA polymerase II, and Rho protein signal transduction. In the CC category, enriched terms were mainly related to cytoplasmic and nuclear structures, including the cytosol, nucleoplasm, histone methyltransferase complex, and nuclear body. Within the MF category, significant enrichment was observed in guanyl–nucleotide exchange factor activity, histone H3K4 mono- and trimethyltransferase activity, transcription coactivator activity, ATP binding, and protein binding. These findings highlight the contribution of PIEZO1-correlated genes to critical regulatory networks controlling transcriptional activity and signal integration in ccRCC.

**Figure 9:**
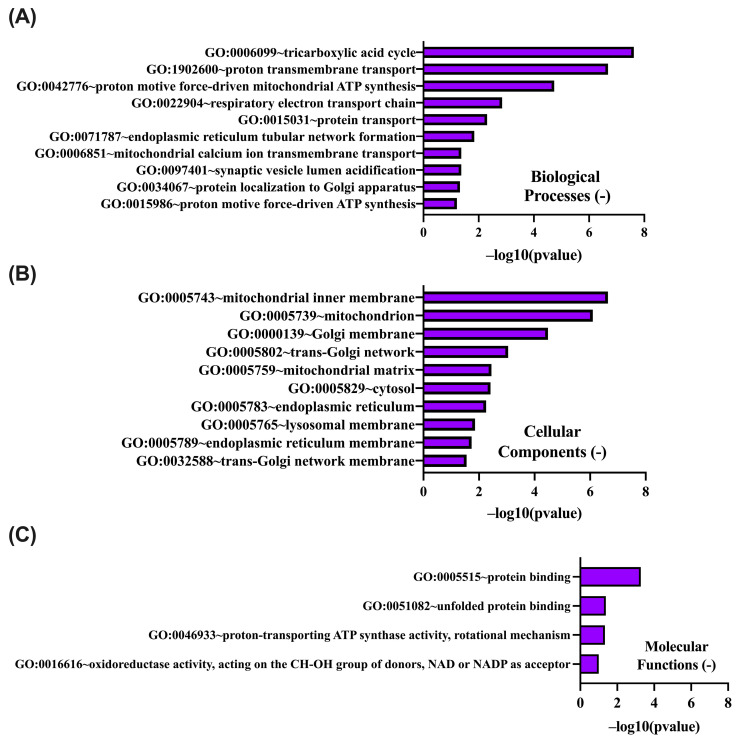
DAVID GO analysis of genes negatively correlated with *PIEZO1*, categorized into BP (**A**), CC (**B**), and MF (**C**). The top 10 GO terms are shown for each category, with *p*-values calculated and ranked based on −log10 (*p*-value).

An analogous GO enrichment analysis was conducted for genes negatively correlated with PIEZO1 expression in ccRCC (Figure 9A–C). Within the BP category, the predominant enrichment was observed for terms associated with mitochondrial energy metabolism, including the tricarboxylic acid cycle, respiratory electron transport chain, and protein translocation. Enriched CC terms were primarily related to mitochondrial, Golgi, and endoplasmic reticulum membranes. In the MF category, significant overrepresentation was noted for protein binding, unfolded protein binding, and ATP synthase activity. These results suggest that genes negatively correlated with PIEZO1 are predominantly involved in mitochondrial metabolism and intracellular transport, which may indicate a functional shift away from bioenergetic maintenance in contexts of elevated PIEZO1 expression.

## 3. Discussion

Clear cell renal cell carcinoma is the most common histological subtype of renal cancer, frequently diagnosed at an advanced clinical stage and associated with poor prognosis [1]. Therapeutic options remain limited, especially in cases of metastatic or therapy-resistant disease [5]. Therefore, there is a growing need to identify novel prognostic markers and molecular therapeutic targets. Due to recent scientific reports regarding the role of PIEZO1 in carcinogenesis, our studies focused on the evaluation of the PIEZO1 protein in ccRCC. PIEZO1 is a mechanosensitive ion channel activated by membrane stretching. It participates in mechanotransduction and plays a role in regulating cell proliferation, adhesion, and migration—processes also essential in carcinogenesis [18]. The role of PIEZO1 in cancer has been demonstrated in colorectal cancer, where its silencing inhibited the expression of HIF-1α and VEGF as reported by Sun et al. [25]. Wang et al. [26] showed that in gastric cancer, increased PIEZO1 expression was associated with enhanced proliferation and inhibition of apoptosis. It also promoted calcium influx. Additionally, increased cell migration and elevated HIF-1α expression were observed [26].

PIEZO1 plays a complex and multifaceted role in cancer development and progression. Elucidating its mechanisms of action and therapeutic potential is essential for advancing effective anticancer strategies. However, despite the growing interest in PIEZO1 in the context of various malignancies, its role in ccRCC remains poorly understood. The present study aimed to address this gap by providing immunohistochemical evidence on the expression of PIEZO1 in ccRCC and evaluating its association with clinicopathological features and the overall survival of patients. Additionally, we incorporated data from TCGA to complement our findings with transcriptomic analyses, offering a broader perspective on the biological relevance of PIEZO1 in ccRCC. To the best of our knowledge, the prognostic relevance of PIEZO1 protein expression had not been previously explored in clear cell renal cell carcinoma (ccRCC). Nonetheless, recent transcriptomic studies have suggested that PIEZO1 mRNA expression may serve as a potential prognostic biomarker in this tumor type [30].

In our study of ccRCC, we observed cytoplasmic expression of PIEZO1 in both tumor cells and adjacent non-tumorous renal tubules. Notably, PIEZO1 expression was more pronounced in peritumoral tubular epithelium than in the tumor tissue itself. This expression pattern contrasts with findings in several other solid malignancies, where PIEZO1 expression is typically upregulated in tumor tissues relative to adjacent normal tissues [20,25,26]. Interestingly, a similar expression trend to our observations was reported by Wu et al. (2022) in lung cancer, where PIEZO1 expression was lower in tumor tissues [31]. In contrast, studies on colorectal adenocarcinoma have demonstrated increased PIEZO1 expression in poorly differentiated tumors compared to well-differentiated ones and to adjacent non-malignant tissues [25]. In hepatocellular carcinoma, PIEZO1 levels were elevated in tumor samples and were associated with more advanced disease stages, consistent with our observations regarding tumor progression [18]. Elevated tumor-specific expression of PIEZO1 has also been reported in prostate cancer [20] and gastric cancer, where PIEZO1 localization was described as both cytoplasmic and membranous [26]. These tumor-specific expression patterns suggest that PIEZO1 may exert context-dependent functions across different cancer types, potentially reflecting diverse regulatory mechanisms and biological roles. In contrast, analysis of the TCGA dataset revealed significantly higher PIEZO1 mRNA levels in tumor tissues compared to non-tumorous counterparts. This discrepancy between transcriptomic and protein-level findings may reflect the influence of multiple layers of gene expression regulation, including post-transcriptional and translational mechanisms that affect mRNA stability, protein synthesis, and degradation. Additionally, such differences may arise from intratumoral heterogeneity and variations within the tumor microenvironment, where distinct cellular subpopulations may differentially express PIEZO1 at the mRNA and protein levels.

In our cohort, a statistically significant association was observed between PIEZO1 protein expression and clinicopathological features, particularly histological grade. However, analysis of TCGA data did not reveal significant correlations between PIEZO1 mRNA levels and clinicopathological parameters. Our findings suggest that elevated PIEZO1 expression may contribute to tumor aggressiveness and progression. The positive correlation with higher tumor grade further supports the potential role of PIEZO1 in promoting tumor growth and advancement. Our findings in ccRCC are consistent with those observed by Qu et al. [23] in gliomas, where higher PIEZO1 expression correlated with higher tumor grade. In addition, in gliomas, PIEZO1 expression was also associated with patient age, histopathological subtype, IDH1 mutation status, and chemotherapy [23].

Kaplan–Meier survival analysis demonstrated significantly reduced overall survival (OS) in ccRCC patients with high PIEZO1 expression, both at the protein and mRNA levels. Moreover, PIEZO1 protein expression remained an independent prognostic factor for poorer OS in multivariate Cox regression analysis. These findings indicate a potentially critical role for PIEZO1 in tumor biology, particularly in relation to its involvement in cellular metabolism and signaling pathways associated with cell proliferation and oxidative stress response. Although high PIEZO1 expression was associated significantly with OS in the overall cohort, this association did not reach statistical significance in patients with stage I disease, indicating that the prognostic value of PIEZO1 may be dependent on tumor stage. In early stage ccRCC, the prognostic impact of PIEZO1 expression may be attenuated by the predominance of other clinical factors such as tumor size, histological grade, and the generally favorable outcome following complete surgical resection. These findings are consistent with the work of Zhu et al. [30], who conducted a bioinformatics investigation of PIEZO1 mRNA expression in RCC across TCGA, MET500, and CPTAC datasets and found poorer OS in patients with higher PIEZO1 expression. Moreover, their results demonstrated that PIEZO1 expression correlates with matrix stiffness and calcium influx and functionally contributes to tumor progression. By responding to extracellular matrix stiffness, PIEZO1 promotes the activation of prooncogenic genes [30]. In our study, Analysis of TCGA data revealed that higher PIEZO1 mRNA expression was associated with reduced overall survival across tumor stages, with the poorest outcomes observed in stages II and III. These findings support the hypothesis that elevated PIEZO1 expression may be linked to increased tumor aggressiveness in ccRCC.

Zhu et al. [30] also demonstrated that *PIEZO1* contributes significantly to the progression of ccRCC. Matrix stiffness was shown to activate *PIEZO1*, leading to calcium influx, calpain activation, and nuclear translocation of YAP. These events collectively enhanced proliferation, epithelial–mesenchymal transition (EMT), and the maintenance of cancer stem-like properties. Notably, silencing *PIEZO1* expression reversed these effects, suggesting its potential as a therapeutic target in ccRCC [30].

Although ccRCC represents the most common histological subtype of kidney cancer, comprehensive molecular characterizations of mechanosensitive signaling pathways in this tumor type remain limited. In this context, cross-cancer comparisons provide valuable insight into conserved and tissue-specific roles of candidate regulators such as *PIEZO1*.

RNA-Seq analysis of the TCGA cohort revealed that *PIEZO1* mRNA expression is significantly upregulated in ccRCC tumor tissues compared to adjacent normal tissues and is associated with poor overall survival, suggesting its potential as a prognostic marker. This observation aligns with the mechanistic framework proposed by Dombroski et al. (2021), who highlighted *PIEZO1* as a key mechanosensor influencing cancer progression through biomechanical signaling, calcium influx, and downstream transcriptional effects [32].

Supporting this concept, our functional enrichment analyses of genes positively correlated with *PIEZO1* revealed strong associations with transcriptional regulatory pathways, particularly those linked to Notch signaling, such as “*NOTCH1* Intracellular Domain Regulates Transcription” and “Signaling by *NOTCH1* in Cancer.” Key hub genes in the associated protein interaction network included *NOTCH1*, *KMT2D*, and *DNMT1*, suggesting that *PIEZO1* may influence epigenetic programming and tumor cell plasticity. In contrast, genes negatively correlated with *PIEZO1* were predominantly involved in mitochondrial metabolism, including oxidative phosphorylation and the TCA cycle, pointing toward a shift in bioenergetics typical of metabolically reprogrammed tumors. These findings support the hypothesis that elevated *PIEZO1* expression in ccRCC may reflect a more aggressive phenotype shaped by altered mechanotransduction and metabolic adaptation [32].

Expanding on the link between *PIEZO1* and NOTCH1 signaling, Chen et al. [33] investigated the role of *PIEZO1* in ischemia/reperfusion-induced acute kidney injury (IR-AKI). Their findings revealed that activation of *PIEZO1* in renal macrophages leads to calcium influx, which subsequently activates calpain signaling. This activation upregulates HIF-1α, which interacts with and stabilizes *NOTCH1*, enhancing its signaling pathway. The enhanced *NOTCH1* signaling promotes M1 macrophage polarization, contributing to inflammation and renal tissue damage. Importantly, myeloid-specific deletion of *PIEZO1* in mice provided protective effects against IR-AKI, highlighting the potential therapeutic value of targeting the *PIEZO1–NOTCH1* axis in acute kidney injuries [33].

Our findings are further supported by recent work by Liang et al. (2025) [34], who demonstrated that *PIEZO1* mRNA expression is significantly elevated in gastric cancer tissues compared to normal gastric mucosa and correlates with advanced TNM stage and poor prognosis. Like our observations in ccRCC, their study identified *PIEZO1* as a potential prognostic biomarker and therapeutic target, highlighting its role in tumor progression. Notably, both studies implicate *PIEZO1* in the activation of transcriptional regulatory networks and in association with aggressive clinicopathological features. While Liang et al. focused on gastric cancer and emphasized correlations with TNM stage and survival, our analysis ccRCC extends this paradigm by revealing a strong link between *PIEZO1* and Notch signaling as well as epigenetic regulation, including key genes such as *NOTCH1*, *KMT2D*, and *DNMT1*. These results collectively suggest that *PIEZO1*-mediated mechanotransduction may drive tumor aggressiveness through both shared and tissue-specific molecular programs, reinforcing its role as a broadly relevant regulator of cancer progression [34].

Consistent with our findings, Poole et al. [19] demonstrated that high *PIEZO1* expression is associated with poorer clinical outcomes in hormone receptor-negative breast cancer patients, particularly those with triple-negative breast cancer. Their study also revealed that tumors with elevated *PIEZO1* expression are enriched in gene signatures related to epithelial–mesenchymal transition, hypoxia, glycolysis, and pro-tumorigenic signaling pathways, while exhibiting decreased infiltration of CD8^+^ and CD4^+^ T cells. These results further support the hypothesis that *PIEZO1* contributes to immune exclusion and enhanced tumor aggressiveness through modulation of the tumor microenvironment [19]. Similarly, Li et al. [35] demonstrated that high *PIEZO1* mRNA expression in primary breast tumors is significantly associated with reduced overall survival, as shown by Kaplan–Meier analysis of transcriptomic data from public datasets. These observations reinforce the association between elevated *PIEZO1* expression and unfavorable clinical outcomes in breast cancer [35].

Duan et al. [36] performed a comprehensive bioinformatics analysis using data from TCGA and the UALCAN database. Their findings revealed that *PIEZO1* expression was significantly lower in NSCLC tissues compared to adjacent normal tissues. Moreover, higher *PIEZO1* mRNA levels were associated with improved overall survival, suggesting a potential tumor-suppressive function in NSCLC. The study also identified miR-942-5p as a negative regulator of *PIEZO1* expression, indicating a possible post-transcriptional regulatory mechanism. In contrast, findings from studies on breast and gastric cancer suggest an opposite trend. In these tumor types, elevated *PIEZO1* expression has been consistently associated with enhanced tumor aggressiveness and poorer clinical outcomes, highlighting the context-dependent role of *PIEZO1* across different malignancies [36].

These observations are further supported by recent pan-cancer analyses, which emphasize the relevance of *PIEZO1* as both a prognostic marker and a modulator of tumor–immune interactions. Wu et al. [31] demonstrated that *PIEZO1* is differentially expressed in a wide range of human malignancies and correlates with immune cell infiltration as well as patient survival. In accordance with their findings, our results confirm that in adrenocortical carcinoma (ACC), elevated *PIEZO1* expression is significantly associated with poorer overall and disease-free survival. Moreover, the strong link between *PIEZO1* expression and stromal signatures identified in our dataset reflects similar associations described in their study, particularly with endothelial cells and cancer-associated fibroblasts [31].

Notably, in our analysis, *NOTCH1* emerged as the top gene positively correlated with *PIEZO1*, supporting a functional interplay between these two mechanosensitive pathways. This association aligns with the findings of Caolo et al. (2019), who demonstrated that in endothelial cells, mechanical activation of *PIEZO1* induces calcium influx, leading to ADAM10-dependent *NOTCH1* cleavage and activation, ultimately driving the expression of Notch target genes and maintaining vascular homeostasis [37].

These observations collectively suggest that *PIEZO1* not only responds to biomechanical cues but also channels them into oncogenic signaling cascades, such as the Notch pathway, thereby promoting tumor cell plasticity, epigenetic reprogramming, and disease progression.

Together, these parallels underscore the potential of *PIEZO1* as a key regulator of the tumor microenvironment in ccRCC and highlight its value as a compelling candidate for further functional validation.

Moreover, it is plausible that PIEZO1 contributes to the adaptation of ccRCC cells to mechanical and metabolic stress within the tumor microenvironment, particularly under hypoxic conditions typical for renal tumors. This aligns with previous evidence that links PIEZO1 to mechanotransduction under low-oxygen environments, where it may enhance tumor cell survival and progression. Further functional studies are needed to clarify whether PIEZO1 exerts a pro-tumorigenic or potentially dual role in ccRCC, depending on tumor stage, differentiation, or microenvironmental pressure.

Future research should aim to elucidate the downstream signaling pathways regulated by PIEZO1 in ccRCC, using gene silencing or overexpression models. It would also be of value to explore the interaction of PIEZO1 with known renal cancer drivers, such as VHL, HIF-1α, or mTOR, and to assess its impact on angiogenesis, metabolic regulation, and immune cell infiltration. Finally, the development of targeted inhibitors or modulators of PIEZO1 could represent a promising direction for therapeutic intervention, particularly in patients with aggressive tumor phenotypes and poor prognosis.

## 4. Materials and Methods

This study retrospectively analyzed archival formalin-fixed, paraffin-embedded (FFPE) tissue material collected from patients with histologically confirmed ccRCC. Tumor samples were obtained during nephrectomy procedures performed between 2009 and 2021 at the Department of Urology and Andrology, Antoni Jurasz University Hospital No. 1 in Bydgoszcz and the Department of Urology and Urological Oncology, Multidisciplinary Hospital of Ludwik Rydygier in Bydgoszcz, Poland. Histopathological verification and classification of tumor samples were conducted at the Department of Clinical Pathomorphology, Collegium Medicum in Bydgoszcz, Nicolaus Copernicus University in Toruń, by two independent pathologists. Out of 114 initially screened cases, 111 were included in the final analysis after excluding samples with insufficient clinical documentation or inadequate tissue quality. Additionally, in 23 patients, adjacent non-malignant renal tissue was available and used as a matched internal control. Tumor grading and staging were assessed in accordance with the WHO/ISUP grading system and the TNM classification criteria. Clinical follow-up data, including patient survival status, were collected from hospital records. Overall survival (OS) was defined as the time from surgery until the last recorded follow-up or death. The end date for survival analysis was set at 8 January 2025.

This study was approved by the Ethics Committee of Collegium Medicum in Bydgoszcz, Nicolaus Copernicus University in Toruń (approval number KB 253/2018).

The construction of tissue macroarrays and the subsequent preparation steps for immunohistochemical staining were performed according to the previously described protocol by Antosik et al. [38]. For the detection of the PIEZO1 antigen, the UltraView detection system (Roche Diagnostics/Ventana Medical Systems, Tucson, AZ, USA) was utilized in combination with a rabbit polyclonal anti-PIEZO1 antibody (cat. no: 15939-1-AP, dilution 1:150; Proteintech, Rosemont, IL, USA), following the manufacturer’s recommendations. Appropriate positive and negative controls were included to validate staining specificity.

Immunohistochemical expression of PIEZO1 was evaluated in a blinded fashion by an experienced pathologist using a light microscope (Olympus BX53; Olympus, Tokyo, Japan) at 20× magnification. The expression level was quantified using the H-score method, which combines staining intensity (0 = negative, 1 = weak, 2 = moderate, 3 = strong) with the percentage of positively stained tumor cells. The final score ranged from 0 to 300, calculated as the sum of the products of staining intensity and the corresponding percentage of cells at each intensity level.

PIEZO1 expression was dichotomized into low- and high-expression groups based on the optimal cutoff value determined using Evaluate Cutpoints software (R version 3.4.1). The optimal cut-off value for PIEZO1 expression was calculated using the cutp method from the survMisc R package (R version 3.4.1). This approach fits a Cox proportional hazards model across all possible cut point values and selects the one that provides the most significant separation in survival between the two resulting groups, based on the log-rank test. The resulting division stratifies patients into low- and high-expression groups in a way that maximizes the prognostic contrast while maintaining clinical interpretability. The established cutoff point was <110 for low-expression and ≥110 for high-expression PIEZO1 in our cohort. Gene expression profiles were retrieved from the UCSC Xena platform in the form of RSEM expected counts normalized using the DESeq2 method. The original transcriptomic data were aligned with the STAR algorithm and quantified via RSEM. The study cohort included 504 cases of clear cell renal cell carcinoma (ccRCC) from The Cancer Genome Atlas (TCGA). Analysis of PIEZO1 gene expression was performed using RNA-seq transcriptomic data obtained from the UCSC Xena Browser (http://xena.ucsc.edu, accessed on 5 February 2025). These data were normalized using the DESeq2 package (v3.21), which applies the median-of-ratios method to account for differences in sequencing depth and RNA composition across samples. A cut-off value of 13.01 was established; samples with expression levels below this threshold were classified as having low PIEZO1 expression, while those with expression levels equal to or above the threshold were categorized as high expression.

To identify genes positively correlated with PIEZO1, the cBioPortal platform (https://www.cbioportal.org; accessed on 15 February 2025) and the TCGA dataset were used to extract the top 50 co-expressed genes. Pathway enrichment analyses and visualizations were carried out using the Reactome Pathway Database (https://reactome.org; accessed on 18 February 2025) and the KEGG Pathway Database (https://www.genome.jp/kegg/pathway.html, accessed on 21 February 2025) to investigate molecular pathways implicated in ccRCC development and progression. PPI networks for the top 50 PIEZO1-co-expressed genes were generated using the STRING database (https://string-db.org; accessed on 2 March 2025) and visualized in Cytoscape software (version 3.10.3, Cytoscape Consortium, San Diego, CA, USA) with the cytoHubba plugin. This analysis was performed using a medium confidence interaction score (0.700), incorporating both 300 positively and 300 negatively correlated genes. Gene Ontology (GO) classification of the co-expressed genes into cellular components (CC), biological processes (BP), and molecular functions (MF) was conducted via the Database for Annotation, Visualization and Integrated Discovery (DAVID; https://david.ncifcrf.gov; accessed on 5 March 2025).

### Statistical Analysis

All statistical analyses and graphical visualizations were conducted using GraphPad Prism (version 8.0; GraphPad Software, San Diego, CA, USA) and RStudio (version 1.3.1093). The normality of continuous variables was evaluated using the Shapiro–Wilk test. Depending on data distribution, either parametric or non-parametric tests were applied as appropriate. Differences in PIEZO1 expression between tumor and adjacent normal tissues were assessed using the Mann–Whitney U test. Associations between categorized PIEZO1 expression and clinicopathological characteristics were examined using the chi-square test or Fisher’s exact test, where applicable. Overall survival (OS) was analyzed using the Kaplan–Meier method, and statistical differences between survival curves were tested using the log-rank test. To identify variables independently associated with OS, univariate and multivariate Cox proportional hazards regression models were employed. Hazard ratios (HRs) and corresponding 95% confidence intervals (CIs) were reported. A backward stepwise approach was used to construct multivariable models, with inclusion and retention thresholds set at *p* < 0.05 and *p* < 0.10, respectively. The assumption of proportional hazards was evaluated graphically through using log(−log) survival plots and analysis of Schoenfeld residuals. Covariates that violated this assumption were modeled as time-dependent variables. All statistical tests were two-sided, and a *p*-value < 0.05 was considered statistically significant.

## 5. Conclusions

Recent progress in cancer biology has brought attention to mechanosensitive channels such as PIEZO1 as potential contributors to tumor progression. This study demonstrates that PIEZO1 expression is significantly altered in ccRCC, with higher protein levels correlating with higher histological grade and shorter overall survival. While our findings support the prognostic relevance of PIEZO1 in renal cancer, the underlying biological mechanisms remain to be fully elucidated. Future research should focus on clarifying whether PIEZO1 operates through shared signaling pathways across tumor types or plays context-specific roles in different cancers. These insights could ultimately inform the development of novel therapeutic strategies targeting mechanotransduction in aggressive renal malignancies.

## Figures and Tables

**Table 1:** PIEZO1 protein and PIEZO1 mRNA expression and its relationship with clinicopathological features of ccRCC patients in our cohort and TCGA cohort. Significant value is in bold.

		PIEZO1 Our Cohort					*PIEZO1* TCGA Cohort		
Variables	Number	Low	High	*p* Value	Variables	Number	Low	High	*p* Value
		*n* = 59	*n* = 52				*n* = 319	*n* = 185	
Age					Age				
≤60	36	21	15	0.5432	≤60	252	155	97	0.4598
>60	75	38	37		>60	252	164	88	
Sex					Sex				
Male	66	31	35	0.1257	Male	324	206	118	0.9232
Female	45	28	17		Female	180	113	67	
Histologic grade					Histologic grade				
G1–G2	95	55	40	**0.0147**	G1–G2	225	145	80	0.5732
G3–G4	16	4	12		G3–G4	271	168	103	
					Gx	8	-	-	
pT status					pT status				
T1	74	40	34	0.3127	T1	251	158	93	0.9184
T2	17	11	6		T2	65	43	22	
T3	20	8	12		T3	178	111	67	
					T4	10	7	3	
pN					pN				
N0	109	58	51	>0.9999	N0	228	150	78	0.7801
N1	2	1	1		N1	15	9	6	
					Nx	261	-	-	
Lymphovascular invasion		(LVI)							
Present	5	3	2	>0.9999					
Absent	106	56	50						
Stage					Stage				
I	74	40	34	0.3127	I	246	155	91	0.8984
II	17	11	6		II	53	36	17	
III	20	8	12		III	124	78	46	
IV	-	-	-		IV	81	50	31	

**Table 2:** Univariable and multivariable analyses of prognostic by Cox regression model in our cohort.

Variable	Univariate Analysis of Own Cohort				Multivariate Analysis of Own Cohort			
	HR	95% CI		*p* Value	HR	95% CI		*p* Value
		Lower	Upper			Lower	Upper	
PIEZO1	2.13	1.14	3.98	0.0182	2.08	1.08	4.01	0.0300
sex	2.66	1.31	5.43	0.0070	2.37	1.14	4.89	0.0200
grade	2.88	1.44	5.75	0.0027	3.22	1.58	6.56	0.0010
pT	3.59	1.84	7.00	0.0002	-	-	-	-
pN	17.17	3.72	79.14	0.0003	-	-	-	-
LVI	6.35	2.23	18.08	0.0005	7.60	2.51	23.06	<0.001

**Table 3:** Top 50 genes positively correlated with *PIEZO1* (Spearman’s coefficient analysis).

*PIEZO1* (+) Correlated Gene	Cytoband	Spearman’s Correlation	*p*-Value	*PIEZO1* (+) Correlated Gene	Cytoband	Spearman’s Correlation	*p*-Value
NLRP1	17p13	0.590	3.05 × 10^−49^	FHOD1	16q22.1	0.528	6.32 × 10^−38^
TECPR1	7q21.3	0.582	1.47 × 10^−47^	CROCC	1p36.13	0.527	9.59 × 10^−38^
NPHP4	1p36.31	0.575	2.95 × 10^−46^	PLEKHG5	1p36.31	0.525	1.89 × 10^−37^
ANKRD11	16q24.3	0.574	6.20 × 10^−46^	MICALL2	7p22.3	0.521	7.30 × 10^−37^
PLCG1	20q12	0.570	2.79 × 10^−45^	MYO9B	19p13.11	0.519	1.49 × 10^−36^
ARHGEF1	19q13.2	0.561	1.19 × 10^−43^	TET3	2p13.1	0.518	2.71 × 10^−36^
PCNT	21q22.3	0.559	2.77 × 10^−43^	FAM219A	9p13.3	0.517	3.97 × 10^−36^
PRDM15	21q22.3	0.558	3.93 × 10^−43^	AATK	17q25.3	0.516	5.59 × 10^−36^
AHDC1	1p36.11-p35.3	0.556	1.04 × 10^−42^	KMT2D	12q13.12	0.514	9.09 × 10^−36^
PKD1	16p13.3	0.556	1.24 × 10^−42^	DENND3	8q24.3	0.514	1.00 × 10^−35^
NCOR2	12q24.31	0.551	9.19 × 10^−42^	CPSF1	8q24.3	0.514	1.15 × 10^−35^
POLE	12q24.33	0.546	5.78 × 10^−41^	DOT1L	19p13.3	0.513	1.62 × 10^−35^
KMT2B	19q13.12	0.544	1.42 × 10^−40^	EML3	11q12.3	0.512	1.85 × 10^−35^
STAB1	3p21.1	0.543	2.09 × 10^−40^	DIP2A	21q22.3	0.510	3.53 × 10^−35^
PLEKHG2	19q13.2	0.541	3.60 × 10^−40^	NOTCH1	9q34.3	0.510	3.60 × 10^−35^
PLCB3	11q13.1	0.541	4.51 × 10^−40^	GRK2	11q13.2	0.510	4.24 × 10^−35^
DDX12P	12p13.31	0.538	1.56 × 10^−39^	HDAC7	12q13.11	0.509	5.34 × 10^−35^
EHBP1L1	11q13.1	0.537	2.04 × 10−^39^	MAP3K6	1p36.11	0.509	5.80 × 10^−35^
CEP164	11q23.3	0.537	2.28 × 10^−39^	PGS1	17q25.3	0.509	5.96 × 10^−35^
DGCR8	22q11.21	0.536	2.59 × 10−^39^	MAP3K12	12q13.13	0.508	7.84 × 10^−35^
BAHCC1	17q25.3	0.535	4.73 × 10^−39^	PITPNM2	12q24.31	0.508	8.52 × 10^−35^
SHANK3	22q13.33	0.534	5.11 × 10^−39^	ZNF335	20q13.12	0.507	9.95 × 10^−35^
DENND4B	1q21.3	0.532	1.34 × 10^−38^	SYNGAP1	6p21.32	0.507	1.14 × 10^−34^
BCL9L	11q23.3	0.531	1.80 × 10−^38^	PPFIA4	1q32.1	0.505	2.12 × 10^−34^
NOD1	7p14.3	0.531	1.93 × 10^−38^	YEATS2	3q27.1	0.505	2.34 × 10^−34^

**Table 4:** Top 50 genes negatively correlated with *PIEZO1* (Spearman’s coefficient analysis).

*PIEZO1* (-) correlated Gene	Cytoband	Spearman’s Correlation	*p*-Value	*PIEZO1* (−) Correlated Gene	Cytoband	Spearman’s Correlation	*p*-Value
TSG101	11p15.1	−0.553	3.23 × 10^−42^	MDH1	2p15	−0.468	3.86 × 10^−29^
SDHD	11q23.1	−0.536	2.66 × 10^−39^	IER3IP1	18q21.1	−0.468	4.23 × 10^−29^
NDUFB5	3q26.33	−0.526	1.16 × 10^−37^	TMEM9B	11p15.4	−0.465	9.92 × 10^−29^
NDFIP1	5q31.3	−0.518	2.37 × 10^−36^	SPCS2P4	1p35.3	−0.464	1.29 × 10^−28^
C18ORF32	18q21.1	−0.516	4.34 × 10^−36^	LAMTOR3	4q23	−0.462	2.39 × 10^−28^
SCOC	4q31.1	−0.513	1.33 × 10^−35^	MED21	12p11.23	−0.461	2.99 × 10^−28^
GHITM	10q23.1	−0.512	1.79 × 10^−35^	VBP1	Xq28	−0.460	4.37 × 10^−28^
NKIRAS1	3p24.2	−0.511	2.80 × 10^−35^	ATP6V1E1	22q11.21	−0.459	5.83 × 10^−28^
ARL1	12q23.2	−0.504	3.75 × 10^−34^	SCAMP1	5q14.1	−0.458	7.79 × 10^−28^
MOCS2	5q11.2	−0.501	8.15 × 10^−34^	HCCS	Xp22.2	−0.456	1.44 × 10^−27^
NDUFA5	7q31.32	−0.493	1.18 × 10^−32^	RTN3	11q13	−0.456	1.60 × 10^−27^
DNAJA2	16q11.2	−0.491	2.54 × 10^−32^	BPGM	7q33	−0.455	1.80 × 10^−27^
ATP6AP2	Xp11.4	−0.486	1.29 × 10^−31^	PAIP1	5p12	−0.455	2.20 × 10^−27^
KGD4	5q13.2	−0.485	1.99 × 10^−31^	UQCRFS1	19q12	−0.454	2.41 × 10^−27^
RNF13	3q25.1	−0.484	2.89 × 10^−31^	SC5D	11q23.3-q24.1	−0.454	2.44 × 10^−27^
ATP5F1A	18q21.1	−0.484	2.96 × 10^−31^	CIAO2A	15q22.31	−0.451	6.20 × 10^−27^
MICU2	13q12.11	−0.480	9.28 × 10^−31^	PLGRKT	9p24.1	−0.450	9.63 × 10^−27^
TATDN3	1q32.3	−0.478	1.64 × 10^−30^	SRP9	1q42.12	−0.446	2.40 × 10^−26^
GABARAPL2	16q23.1	−0.476	2.92 × 10^−30^	IDH3A	15q25.1	−0.445	3.29 × 10^−26^
CHP1	15q15.1	−0.475	5.32 × 10^−30^	SYS1	20q13.12	−0.444	4.95 × 10^−26^
ARL6IP1	16p12.3	−0.473	9.97 × 10^−30^	LSM3	3p25.1	−0.444	4.97 × 10^−26^
MRPS35	12p11.22	−0.472	1.09 × 10^−29^	SUCLG1	2p11.2	−0.443	6.48 × 10^−26^
MKKS	20p12.2	−0.472	1.30 × 10^−29^	PDHB	3p14.3	−0.434	7.79 × 10^−25^
SKP1	5q31.1	−0.470	2.04 × 10^−29^	COPS4	4q21.22	−0.434	8.47 × 10^−25^
ATP5PB	1p13.2	−0.470	2.54 × 10^−29^	PLEKHA3	2q31.2	−0.433	9.35 × 10^−25^

## Data Availability

The datasets generated during and/or analyzed during the current study are available from the corresponding author on reasonable request.
